# Changing Mortality Profile among HIV-Infected Patients in Rio de Janeiro, Brazil: Shifting from AIDS to Non-AIDS Related Conditions in the HAART Era

**DOI:** 10.1371/journal.pone.0059768

**Published:** 2013-04-05

**Authors:** Beatriz Grinsztejn, Paula M. Luz, Antonio G. Pacheco, Desiree V. G. Santos, Luciane Velasque, Ronaldo I. Moreira, Maria Regina C. Guimarães, Estevão P. Nunes, Alberto S. Lemos, Sayonara R. Ribeiro, Dayse P. Campos, Marco A. A. Vitoria, Valdilea G. Veloso

**Affiliations:** 1 Instituto de Pesquisa Clínica Evandro Chagas, Fundação Oswaldo Cruz, Rio de Janeiro, Brasil; 2 Programa de Computação Científica, Fundação Oswaldo Cruz, Rio de Janeiro, Brasil; 3 Departamento de Matemática e Estatística, Universidade Federal do Estado do Rio de Janeiro, Rio de Janeiro, Brasil; 4 HIV Department, World Health Organization, Geneva, Switzerland; Hopital Bichat Claude Bernard, France

## Abstract

**Introduction:**

We describe temporal trends in the mortality rates and factors associated with AIDS and non-AIDS related mortality at the Evandro Chagas Clinical Research Institute (IPEC), Oswaldo Cruz Foundation (FIOCRUZ).

**Methods:**

Adult patients enrolling from 1986 through 2009 with a minimum follow up of 60 days were included. Vital status was exhaustively checked using patients’ medical charts, through active contact with individuals and family members and by linkage with the Rio de Janeiro Mortality database using a previously validated algorithm. The CoDe protocol was used to establish the cause of death. Extended Cox proportional hazards models were used for multivariate modeling.

**Results:**

A total of 3530 individuals met the inclusion criteria, out of which 868 (24.6%) deceased; median follow up per patient was 3.9 years (interquartile range 1.7–9.2 years). The dramatic decrease in the overall mortality rates was driven by AIDS-related causes that decreased from 9.19 deaths/100PYs n 1986–1991 to 1.35/100PYs in 2007–2009. Non-AIDS related mortality rates remained stable overtime, at around 1 death/100PYs. Immunodeficiency significantly increased the hazard of both AIDS-related and non-AIDS-related causes of death, while HAART use was strongly associated with a lower hazard of death from either cause.

**Conclusions:**

Our results confirm the remarkable decrease in AIDS-related mortality as the HIV epidemic evolved and alerts to the conditions not traditionally related to HIV/AIDS which are now becoming more frequent, needing careful monitoring.

## Introduction

Highly-active antiretroviral therapy (HAART) had a dramatic impact on the AIDS epidemic; in particular, it has greatly changed the mortality profile for human immunodeficiency virus (HIV)-infected individuals. In the pre-HAART era there was a clear predominance of AIDS-related conditions whereas, currently, the mortality profile is more diverse, including cardiovascular diseases, non-AIDS-related-malignancies and other end-organ diseases such as end-stage liver and renal diseases [1], [2], [3]. Indeed, as survival increases, so does the likelihood that diseases other than those related to AIDS will incur among HIV-infected individuals. Within this changing scenario, the surveillance of causes of death in HIV-infected patients is critically important to understand the changing epidemiology of HIV disease, to improve HIV clinical management and the management of co-morbidities, and to identify the potential side effects of antiretroviral therapy.

In Brazil, mono and dual antiretroviral therapy started being provided in the early 1990s and HAART has been made available since 1996. Local studies have shown an increased survival of HIV-infected individuals and, more recently, an increased frequency of non-AIDS-related causes of death among HIV-infected individuals [3], [4]. Our objective was to describe the overall and the AIDS and non-AIDS related mortality rates, their temporal trends and the factors associated with the causes of death in a reference center for HIV care, the Evandro Chagas Clinical Research Institute (IPEC), Oswaldo Cruz Foundation (FIOCRUZ).

## Methods

### Study Site

IPEC is a national reference center for infectious diseases and has been a reference center for care, research, and training related to HIV/AIDS since 1986. The AIDS program at IPEC is one of the largest providers of primary, specialty, and tertiary care for HIV infected individuals and AIDS patients in Rio de Janeiro State.

A longitudinal observational clinical database has been maintained on patients receiving HIV care at IPEC. Cohort procedures have been described and results published [5], [6], [7]. Briefly, data are updated regularly using outpatient and inpatient clinical documentation and laboratory testing results. Prescription of antiretroviral therapy (drug, dates of use, and dose) is documented by the medical provider and support staff in the clinical records. Trained abstractors record the information onto standardized forms for processing.

### Study Population and Outcome Definition

The study population included adult patients (age ≥18 years) who enrolled in the IPEC cohort and had a minimum follow up of 60 days, from September 12, 1986 (the date of the first enrollee) through October 30, 2009 (to allow the minimum of 60 days of follow up until December 31, 2009). End of follow up was defined as the earlier of death or December 31 2009 when administrative censoring was applied. Information regarding vital status was exhaustively checked up until December 31 2009 using the patients’ medical charts, through active contact with individuals and family members and by linkage with the Rio de Janeiro Mortality database using a previously validated algorithm [8].

For all deaths that occurred in the study period, the CoDe (“Coding of Death in HIV”) method (http://www.cphiv.dk/CoDe/) was used to revise near death patient information. The CoDe method is a uniform coding system that includes detailed data collection on the causes of death and contributing factors and a centralized review process of the collected data. The Portuguese translated CoDe case report forms were completed by two physicians specialized in HIV care. The central review process followed the CoDe guidelines and was performed independently by two qualified reviewers also specialized in HIV care. If agreement on the immediate and underlying causes of death was reached, the cause of death was established. If there was disagreement between the two reviewers, the specific case was referred to one additional reviewer who reviewed the CoDe case report and the CoDe review forms and then established the cause of death.

The cause of death was given by the underlying cause of death. Groups of causes of death were defined based on the categories present in the CoDe review form as follows: AIDS-infection (CoDe 01.1), AIDS-malignancy (CoDe 01.2), AIDS-not specified (CoDe 01), non-AIDS infection (CoDe 02), liver-related (CoDe 03), non-AIDS malignancy (CoDe 04), diabetes mellitus (CoDe 05), pancreatitis (CoDe 06), toxicity (CoDes 07, 14), cardiovascular (CoDes 08, 09, 24), primary pulmonary hypertension (CoDe 11), lung embolus (CoDe 12), respiratory disease (CoDes 13, 25), renal failure (CoDe 15), violence (CoDe 16), psychiatric (CoDe 17, 22), substance abuse (CoDe 19), hematological disease (CoDe 20), endocrine disease (CoDe 21), central nervous system (CNS) disease (CoDe 23), digestive system disease (CoDe 10, 26), skin and motor system disease (CoDe 27), urogenital disease (CoDe 28), obstetric complication (CoDe 29), congenital disorders (CoDe 30) and unknown (CoDe 90, 91, 92). Of the above mentioned causes of death, those with two deaths or less were combined in the “Other causes” group.

### Definition of Variables of Interest

Age at enrolment was calculated as the difference between date of birth and date of enrolment. Age at the end of follow up was calculated as the difference between date of birth and end of follow up as defined above. Race/ethnicity was based on the provider’s report and was categorized into white and non-white. Education was self-reported and categorized according to the number of years in school into three groups: ≤5 years, 6–9 years, and ≥10 years. Presumed HIV exposure category were self-reported and categorized hierarchically into injection drug use (IDU, irrespective of sexual practices), men who have sex with men (MSM, includes bisexuals), heterosexual and other/unknown. Baseline CD4 cell count was defined within a window of 180 days of enrolment, and, if more than one measurement was available, the value closest to the date of enrolment was chosen. HAART use was defined as the use of at least three antiretroviral drugs: two nucleoside reverse transcriptase inhibitor and either one protease inhibitor or one non-nucleoside reverse transcriptase inhibitor, and categorized into those who, at any given point in time, used HAART for more than 12 weeks, and those who never used HAART (never used or used for 12 weeks or less). AIDS-diagnosis (CDC 1993 definition) at baseline was defined by the occurrence of an AIDS-defining illness at or before cohort enrolment.

### Statistical Analysis

Descriptive statistics for demographic and clinical variables were compared for four groups defined as a function of vital status and category of underlying cause of death (alive, and death due to non-AIDS related, AIDS-related and unknown causes). Kruskal-Wallis and Chi-squared tests were used to access differences among groups for continuous and categorical variables, respectively. Incidence rates of death per 100 person-years were calculated for all causes, and for AIDS-related and non-AIDS-related causes of death. For the calculation of the rates, the study period was divided into three-year periods, except for the first period, to allow for a more precise description and assessment of temporal trends. Temporal trends over periods were tested by exact mid-P values calculation.

To explore the factors associated with AIDS or non-AIDS related causes of death, we used extended Cox proportional hazards models, accounting for competing risks, as described elsewhere [4]. For this analysis, we restricted the data to patients who enrolled the cohort after HAART was widely available in Brazil (i.e. from 1997 onwards, referred to as 1997+). The reasoning behind this restriction was the fact that we did not want to show the association of HAART use for all patients, that is, for those who did and did not have HAART available to them. The inclusion of a variable mixing pre-HAART and HAART eras resulted, as expected, in a lack of hazards proportionality. Similarly, the inclusion of time-updated CD4 counts also resulted in a lack of hazards proportionality. Thus, we chose the time when HAART was made widely available (from 1997 onwards) as the partition point. In a sensitivity analysis, we also ran the models with the full data, stratifying for the variables that were not proportional over time (i.e., HAART use and time-updated CD4 counts). The latter approach is also valid although it does not allow one to explore the effect of the stratification variables.

### Ethical Approval

This study was conducted according to the principles expressed in the Declaration of Helsinki. This study was approved by the ethics committee of the Evandro Chagas Clinical Research Institute, Oswaldo Cruz Foundation. Participants provided written informed consent.

## Results

During the study period, a total of 3530 individuals met the inclusion criteria, out of which 868 (24.6%) deceased. Some demographic and clinical characteristics were significantly different across the groups alive, death due to non-AIDS related and death due to AIDS-related causes of deaths (Table 1), such as gender (65%, 68.9% and 75.1% male, respectively), baseline CD4 counts (323, 264.5 and 124.5 cells/mm3, respectively), HAART use (74.7%, 53.8% and 28.3%, respectively), AIDS diagnosis at baseline (41.2%, 68,4% and 82.2%, respectively) and median follow-up time (4.6, 2.9 and 2 years, respectively). Median age at enrollment and at the end of follow-up showed contrasting patterns; while the former was similar and lower for those alive and who died of AIDS-related causes (34.9 and 34.7 years, respectively) when compared to those who died of non-AIDS-related causes (38.1 years), the latter was similar and higher for those alive and who died of non-AIDS-related causes (42.8 years for both) when compared to those who died of AIDS-related causes (38.1 years). Also, a significantly higher percentage of individuals alive completed 10 or more years in school compared to those who died, irrespective of the cause of death. The highest percentage of individuals reporting injection drug use was observed among those who died of non-AIDS related causes of death (8.1%). Race distribution was homogeneous across groups. The death of unknown cause group was too small to draw consistent conclusions.

**Table 1:** Demographic and clinical characteristics of the study population by vital status and cause of death.

	Alive	Non-AIDS-related death	AIDS-related death	Death unknown	p-value
Total	2662	209	639	20	
Age at enrolment (years)					
Median (IQR)	34.9 (28.5,42)	38.1 (31.1,46.5)	34.7 (28.7,42)	30.3 (27.4,39.8)	<0.001
[18,30)	829 (31.1)	47 (22.5)	187 (29.3)	10 (50)	
[30,40)	1001 (37.6)	72 (34.4)	256 (40.1)	5 (25)	
[40,50)	604 (22.7)	51 (24.4)	144 (22.5)	4 (20)	<0.001
[50,60)	185 (6.9)	28 (13.4)	41 (6.4)	1 (5)	
[60+	43 (1.6)	11 (5.3)	11 (1.7)	0 (0)	
Age at end of follow up (years)					
Median (IQR)	42.8 (35,49.9)	42.8 (36.7,51.6)	38.1 (32.1,45.1)	38 (31.5,49.3)	<0.001
[18,30)	326 (12.2)	21 (10)	119 (18.6)	4 (20)	
[30,40)	763 (28.7)	55 (26.3)	253 (39.6)	8 (40)	
[40,50)	914 (34.3)	79 (37.8)	187 (29.3)	3 (15)	<0.001
[50,60)	494 (18.6)	33 (15.8)	62 (9.7)	4 (20)	
[60+	165 (6.2)	21 (10)	18 (2.8)	1 (5)	
Gender					<0.001
Female	931 (35)	65 (31.1)	159 (24.9)	5 (25)	
Male	1731 (65)	144 (68.9)	480 (75.1)	15 (75)	
Race					0.086
White	1500 (56.5)	118 (56.7)	359 (56.4)	17 (85)	
Non-white	1156 (43.5)	90 (43.3)	277 (43.6)	3 (15)	
Education (years in school)					<0.001
≤5	654 (24.8)	66 (32.2)	191 (31.5)	6 (33.3)	
6–9	645 (24.5)	69 (33.7)	153 (25.2)	4 (22.2)	
≥10	1333 (50.6)	70 (34.1)	262 (43.2)	8 (44.4)	
HIV exposure category					<0.001
Heterosexual	1286 (48.3)	94 (45)	223 (34.9)	8 (40)	
MSM	952 (35.8)	56 (26.8)	271 (42.4)	8 (40)	
IDU	44 (1.7)	17 (8.1)	28 (4.4)	1 (5)	
Unknown/Other	380 (14.3)	42 (20.1)	117 (18.3)	3 (15)	
Baseline CD4 cell count (cells/mm3)					
Median (IQR)	323 (140,530)	264.5 (89.5,430.2)	124.5 (50.5,299.8)	537 (213.8,857.5)	<0.001
≤100	430 (19.1)	30 (25.9)	114 (42.9)	2 (20)	
101–200	317 (14.1)	19 (16.4)	51 (19.2)	1 (10)	
201–350	460 (20.5)	28 (24.1)	44 (16.5)	0 (0)	<0.001
351–500	409 (18.2)	18 (15.5)	22 (8.3)	2 (20)	
>500	631 (28.1)	21 (18.1)	35 (13.2)	5 (50)	
HAART use*					<0.001
No	674 (25.3)	96 (46.2)	458 (71.7)	14 (70)	
Yes	1988 (74.7)	112 (53.8)	181 (28.3)	6 (30)	
AIDS diagnosis					<0.001
No	1566 (58.8)	66 (31.6)	114 (17.8)	13 (65)	
Yes	1096 (41.2)	143 (68.4)	525 (82.2)	7 (35)	
Follow-up (years)					<0.001
Median (IQR)	4.6 (2.2,10.5)	2.9 (0.8,7.6)	2 (0.5,4.7)	4.9 (3.4,8.6)	

Deaths were categorized into non-AIDS-related, AIDS-related, and of unknown cause. Columns provide number (percentage) unless otherwise specified.

IQR: interquartile range, MSM: men who have sex with men, IDU: injection drug use, HAART: highly active antiretroviral treatment.

* Defined as use of HAART for at least 12 weeks.

The remarkable changes in the relative frequency of causes of death are shown in Table 2. Overall, 73.6% (639/868) of all deaths were due to AIDS-related causes while 26.4% (229/868) were due to non-AIDS related causes. Non-AIDS-related causes of death increased from 13.3% in 1986–1991 to as much as 38.3% in 2007–2009, while AIDS-related causes, for these same periods, decreased from 86.7% to 61.7%.

**Table 2:** Number (percent) of causes of death overall, AIDS and non-AIDS related and by specific causes by calendar periods.

	1986–91	1992–94	1995–97	1998–00	2001–03	2004–06	2007–09	Total
**Total**	113 (100)	169 (100)	127 (100)	86 (100)	99 (100)	125 (100)	149 (100)	868 (100)
**All deaths by major groups**								
**AIDS related (CoDe 01.1, 01.2, 01)**	98 (86.7)	150 (88.8)	100 (78.7)	64 (74.4)	65 (65.7)	70 (56)	92 (61.7)	639 (73.6)
**Non-AIDS related (CoDe 02–30)**	12 (10.6)	17 (10.1)	25 (19.7)	19 (22.1)	31 (31.3)	52 (41.6)	53 (35.6)	209 (24.1)
**Unknown (90, 91, 92)**	3 (2.7)	2 (1.2)	2 (1.6)	3 (3.5)	3 (3)	3 (2.4)	4 (2.7)	20 (2.3)
**Total**	113 (100)	169 (100)	127 (100)	86 (100)	99 (100)	125 (100)	149 (100)	868 (100)
**All deaths by specific causes**								
**AIDS-infection (CoDe 01.1)**	75 (66.4)	95 (56.2)	65 (51.2)	44 (51.2)	39 (39.4)	42 (33.6)	52 (34.9)	412 (47.5)
**AIDS-malignancy (CoDe 01.2)**	2 (1.8)	12 (7.1)	3 (2.4)	2 (2.3)	6 (6.1)	12 (9.6)	16 (10.7)	53 (6.1)
**AIDS-not specified (CoDe 01)**	21 (18.6)	43 (25.4)	32 (25.2)	18 (20.9)	20 (20.2)	16 (12.8)	24 (16.1)	174 (20.0)
**Non-AIDS infection (CoDe 02)**	5 (4.4)	3 (1.8)	7 (5.5)	0 (0)	5 (5.1)	8 (6.4)	17 (11.4)	45 (5.2)
**Liver-related (CoDe 03)**	0 (0)	0 (0)	2 (1.6)	2 (2.3)	4 (4.0)	5 (4.0)	3 (2.0)	16 (1.8)
**Non-AIDS malignancy (CoDe 04)**	0 (0)	2 (1.2)	0 (0)	2 (2.3)	2 (2.0)	9 (7.2)	9 (6.0)	24 (2.8)
**Diabetes mellitus (CoDe 05)**	0 (0)	0 (0)	1 (0.8)	1 (1.2)	0 (0)	1 (0.8)	1 (0.7)	4 (0.5)
**Pancreatitis (CoDe 06)**	0 (0)	0 (0)	0 (0)	3 (3.5)	3 (3.0)	1 (0.8)	1 (0.7)	8 (0.9)
**Toxicity (CoDe 07, 14)**	0 (0)	1 (0.6)	1 (0.8)	0 (0)	1 (1.0)	1 (0.8)	3 (2.0)	7 (0.8)
**Cardiovascular (CoDe 08, 09, 24)**	2 (1.8)	3 (1.8)	1 (0.8)	2 (2.3)	5 (5.1)	13 (10.4)	8 (5.4)	34 (3.9)
**Respiratory disease (CoDe 13, 25)**	1 (0.9)	2 (1.2)	1 (0.8)	1 (1.2)	1 (1.0)	2 (1.6)	1 (0.7)	9 (1.0)
**Renal failure (CoDe 15)**	0 (0)	1 (0.6)	1 (0.8)	0 (0)	0 (0)	3 (2.4)	2 (1.3)	7 (0.8)
**Violence (CoDe 16)**	0 (0)	1 (0.6)	6 (4.7)	5 (5.8)	5 (5.1)	5 (4.0)	3 (2.0)	25 (2.9)
**Substance abuse (CoDe 19)**	0 (0)	0 (0)	0 (0)	1 (1.2)	0 (0)	2 (1.6)	0 (0)	3 (0.3)
**Hematological disease (CoDe 20)**	0 (0)	0 (0)	1 (0.8)	1 (1.2)	1 (1.0)	1 (0.8)	1 (0.7)	5 (0.6)
**Central nervous system disease (CoDe 23)**	3 (2.7)	0 (0)	2 (1.6)	0 (0)	0 (0)	0 (0)	0 (0)	5 (0.6)
**Digestive disease (CoDe 10, 26)**	1 (0.9)	3 (1.8)	2 (1.6)	0 (0)	0 (0)	0 (0)	2 (1.3)	8 (0.9)
**Other causes** *	0 (0)	1 (0.6)	0 (0)	1 (1.2)	4 (4.0)	1 (0.8)	2 (1.3)	9 (1.0)
**Unknown (90, 91, 92)**	3 (2.7)	2 (1.2)	2 (1.6)	3 (3.5)	3 (3.0)	3 (2.4)	4 (2.7)	20 (2.3)
**Total**	113 (100)	169 (100)	127 (100)	86 (100)	99 (100)	125 (100)	149 (100)	868 (100)

* Causes of death for which less than 2 deaths were observed were grouped in the “Other causes” group (CoDe 11, 12, 17, 21, 22, 27–30).

Among the specific causes, the percentage of deaths due to AIDS-infection decreased from 66.4% in 1986–1991 to 34.9% in 2007–2009. The top three causes of death within the AIDS-infection group were tuberculosis (149/412, 36%), cytomegalovirus (49/412, 12%), and cryptococcosis (46/412, 11%). In contrast, the percentage of deaths due to AIDS-malignancy increased from 1.8% in 1986–1991 to 10.7% in 2007–2009. The top two causes of death within the AIDS-malignancy group were Kaposi’s sarcoma (22/53, 42%) and non-Hodgkin lymphoma (18/53, 34%).

Non-AIDS infection was the most frequent non-AIDS cause of death with 5.2% (45/868) of all deaths (Table 2). Sepsis (31/45, 69%) and pneumonia (7/45, 16%) accounted for the great majority of causes of death within the non-AIDS infection group. Cardiovascular disease was the second most frequent non-AIDS cause of death with 3.9% of all deaths (34/868), with the most frequent diagnoses being myocardial infarction (14/34, 41%), stroke (5/44, 15%), and heart failure (5/44, 15%). Non-AIDS-malignancies accounted for 2.8% of all deaths (24/868) with lung cancer (4/24, 17%) as the most frequently reported cause of death.

**Figure 1:**
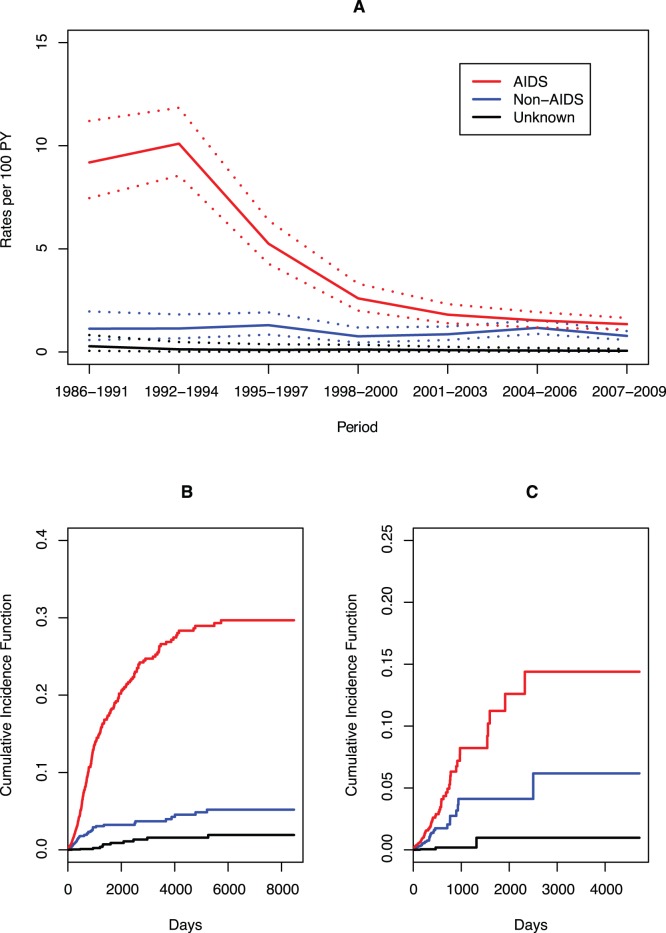
Mortality rates and Cumulative Incidence Functions for AIDS-related, non-AIDS related and unknown causes of death. Mortality rates for AIDS-related, non-AIDS related, and unknown causes of death over calendar periods (A) and Cumulative Incidence Functions for AIDS-related, non-AIDS related and unknown causes of death for the entire cohort (B) and for those enrolling from 1997 onwards (C).

Mortality rates from all causes decreased remarkably from over 10 deaths/100 person-years (PYs), to almost 2/100PYs during the study period (Table 3). This decrease was driven by AIDS-related causes of death, which also decreased in a similar fashion, from 9.19 deaths/100PYs to 1.35/100 PYs during the same period (Table 3 and Figure 1A). Both decreases were statistically significant when the first period was used as the reference category (Table 3). Non-AIDS related mortality rates, on the other hand, remained relatively stable during the study period, at around 1 death/100 PYs (Table 3 and Figure 1A).

**Table 3:** Mortality rates overall, and for AIDS-related and non-AIDS related causes of death over calendar periods.

	Overall			Non-AIDS-related			AIDS-related			
Period	Deaths	Rate	p-value	Deaths	Rate	p-value	Deaths	Rate	p-value	PYs
1986–1991	113	10.6	Reference	12	1.13	Reference	98	9.19	Reference	1066.39
1992–1994	170	11.37	0.565	17	1.14	0.987	151	10.1	0.47	1495.57
1995–1997	128	6.65	<0.001	25	1.3	0.696	101	5.25	<0.001	1923.42
1998–2000	87	3.48	<0.001	19	0.76	0.293	65	2.6	<0.001	2501.6
2001–2003	96	2.76	<0.001	30	0.86	0.437	63	1.81	<0.001	3473.46
2004–2006	125	2.77	<0.001	53	1.17	0.921	69	1.53	<0.001	4515.32
2007–2009	149	2.18	<0.001	53	0.78	0.253	92	1.35	<0.001	6835.62
Total	868	3.98	–	209	0.96	–	639	2.93	–	21811.38

The cumulative incidence functions for AIDS-related, non-AIDS related and unknown causes of death are shown in the figure for the entire cohort (Figure 1B) and for the subset 1997+ (Figure 1C). The sinusoid shape of the curves for AIDS-related causes of death clearly show that the highest acceleration of risk accumulation occurred in the beginning of the follow-up until a steady value is reached. This pattern is observed for the entire cohort (Figure 1B, red curve) and the 1997+ subset (Figure 1C, red curve). For non-AIDS related causes, the curves present a more familiar log-function shape. These patterns reflect the dramatic decrease of AIDS-related causes as opposed to the relatively steady behavior of non-AIDS related causes of death.

Table 4 gives the results of the competing risks Cox models. Lower CD4 cell counts and AIDS diagnosis at baseline significantly increased the hazard of AIDS-related and non-AIDS-related causes of death, although the magnitude of the effect of both factors was much higher for AIDS-related causes of death. HAART use, on the other hand, was strongly associated with a lower hazard of death from either cause. Older age and lower education significantly increased the hazards for non-AIDS related causes of death, but not for AIDS-related causes of death. Indeed, the hazard for non-AIDS related causes of death as a function of age shows a clear dose-response relationship, increasing from a non-significant hazard ratio of 1.44 for those aged 30–39 years compared to the <30 years reference category, to a highly significant hazard ratio of 6.41 for those aged 60 years or more.

**Table 4:** Adjusted hazard ratios (HR) and 95% confidence intervals (95%CI) for factors associated with non-AIDS-related and AIDS-related causes of death estimated using extended Cox proportional hazards models, accounting for competing risks.

		Non-AIDS related			AIDS-related		
Variable	Category	HR	95% CI	p-value	HR	95% CI	p-value
Age	−30)	Ref.			Ref.		
	[30,40)	1.44	(0.79–2.61)	0.24	1.36	(0.89–2.08)	0.15
	[40,50)	1.54	(0.79–2.98)	0.2	1.44	(0.91–2.28)	0.12
	[50,60)	4.27	(2.13–8.57)	<0.001	1.37	(0.74–2.53)	0.31
	[60+	6.41	(2.31–17.77)	<0.001	1.55	(0.46–5.21)	0.47
Gender	Female	Ref.			Ref.		
	Male	1.02	(0.65–1.6)	0.94	1.18	(0.83–1.67)	0.35
Race	White	Ref.			Ref.		
	Non-white	1.03	(0.67–1.6)	0.89	0.85	(0.61–1.18)	0.34
Education (years in school)	≤5	2.21	(1.23–3.97)	0.01	1.25	(0.84–1.86)	0.28
	6–9	3.17	(1.82–5.52)	<0.001	1.23	(0.83–1.81)	0.3
	≥10	Ref.			Ref.		
IDU	Yes	2.88	(0.88–9.39)	0.08	0.75	(0.18–3.09)	0.69
	No	Ref.			Ref.		
HAART use*	Yes	0.10	(0.06–0.18)	<0.001	0.04	(0.03–0.06)	<0.001
	No	Ref.			Ref.		
AIDS diagnosis	Yes	2.26	(1.34–3.82)	<0.001	8.25	(4.75–14.34)	<0.001
	No	Ref.			Ref.		
CD4 cell count**	[0,100]	16.12	(7.13–36.45)	<0.001	67.84	(31.91–144.23)	<0.001
	(100,200]	4.74	(1.82–12.34)	<0.001	31.6	(14.64–68.19)	<0.001
	(200,350]	5.25	(2.37–11.63)	<0.001	5.96	(2.65–13.41)	<0.001
	(350,500]	5.55	(2.61–11.81)	<0.001	2.23	(0.88–5.65)	0.09
	(500+	Ref.			Ref.		

IDU: injection drug use, HAART: highly active antiretroviral treatment, ADI: AIDS defining illness.

* Defined as HAART use for at least 12 weeks.

** CD4 cell counts (cells/mm3) were modeled as a time-updated variable.

## Discussion

We retrospectively assigned a defined cause of death to 97.7% of the 868 deaths occurring among the 3530 individuals followed at IPEC from 1986 to 2009. We showed that mortality rates from AIDS-related causes of death dramatically decreased over time from 9.19 deaths/100 PYs in 1986–1991 to 1.35 deaths/100 PYs in 2007–2009. In contrast, mortality rates from non-AIDS-related causes showed no trend over time, oscillating at 1 death/100 PYs. Moreover, remarkable changes in the relative frequency of AIDS-related and non-AIDS-related causes of death were observed with AIDS-related causes decreasing from 87% to 62% (and, complementarily, non-AIDS-related causes of death increasing from 13% to 38%) in the periods 1986–1991 and 2007–2009, respectively. These results are in agreement with reports from Brazil and other countries that show a remarkable decrease in AIDS-related causes of death as the HIV epidemic progressed through time and corroborates the observation that conditions that are not traditionally related to HIV/AIDS are now becoming relatively more frequent, as reflected in our findings [2], [3], [4], [9], [10].

We found that patients who died from AIDS-related causes of death had poorer immunological profile at cohort enrollment compared to those who remained alive or died of non-AIDS-related causes of death. Median CD4 cell counts at baseline for patients who died of AIDS-related infections and AIDS-related malignancies were, respectively, 117 and 146 cells/mm3, as opposed to 296 cells/mm3 for patients who died of non-AIDS related causes. Additionally, a significantly higher percentage of those who died of AIDS related causes had AIDS diagnosis at enrollment. Indeed, AIDS diagnosis at enrollment and lower CD4 cell count were significantly associated with a higher hazard of death from AIDS-related causes in the multivariate analysis. In previous analyses, we have shown that late presentation to care is a major problem in our setting [5], [7]. Individuals who present late are at increased risk of AIDS-related clinical events and death and are more likely to present with multiple conditions within a short period of time [11], [12]. Other studies have shown that prior AIDS defining illness was also found to be associated with AIDS-related mortality even among patients who attained CD4 cell counts >500 cells/mm3, underlining the importance of early diagnosis and treatment of HIV infection [13] and early initiation of antiretroviral treatment.

We also found that HAART use was significantly associated with a lower hazard of death from AIDS-related and non-AIDS related causes. In a scenario where antiretroviral treatment has been shown to dramatically reduce mortality rates attributable to HIV-infection and where improvements in antiretroviral treatment have further reduced mortality rates among HIV-infected individuals, the significant association of lower CD4 cell counts with higher rates of death due to AIDS and non-AIDS related causes supports the numerous arguments for earlier initiation of antiretroviral treatment [14], [15], [16], [17]. It is also reassuring that no evidence of an increased risk for both all-cause and non-AIDS-related deaths with long-term cumulative HAART exposure was reported in a recent publication from the Eurosida cohort [18].

AIDS-infection remained the most frequent cause of death throughout the 25 years evaluated in the present study. This finding has also been reported in several other cohorts from the developed and developing world [4], [19], [20]. Among AIDS-related infections, tuberculosis was the number one cause of death. In countries where tuberculosis is a major cause of death, as is our setting, the need to early diagnose and treat HIV infection is paramount [21].

In the adjusted analysis, we found that older age, lower education, and lower CD4 cell counts significantly increased the hazard of death from non-AIDS related causes. Compared to those who died from AIDS-related conditions, patients who died of non-AIDS-related causes of death were older at cohort enrolment and at the time of death. An aging HIV-infected population presents new challenges that include the monitoring and handling of drug interactions resulting from the treatment of chronic co-morbid conditions and HIV infection.

Among non-AIDS-related deaths, non-AIDS-infections were the most frequent cause of death. We have previously shown that infections play a major role in early mortality [5]. Poor economic conditions may predispose individuals to community-acquired bacterial infections. Hence, strategies to increase vaccination coverage against Streptococcus pneumoniae and influenza virus should be implemented. In Brazil, as a significant fraction of patients die in the hospital, sepsis emerges as a frequent end-of-life complication [22], [23]. Patients with HIV are at increased risk for nosocomial infections because of immunosuppression, frequent invasive procedures and multispectral antibiotic administration. The development of better preventive and treatment options for nosocomial/health care-associated infections in this population may be effective both clinically and financially.

Cardiovascular disease was the second most frequent cause of death among non-AIDS related causes. Similar to several other HIV-associated co-morbidities, cardiovascular disease stems from the interplay of multiple risk factors, including both traditional risk factors and those specific to HIV infection, such as impaired immune function disease [24], [25], [26]. Improvement of immunologic function is likely to be an important component of cardiovascular disease prevention for patients with HIV-infection.

Our results also show that the proportion of deaths attributable to both AIDS-malignancy and non-AIDS-malignancy increased over time. The most common AIDS-related malignancies were Kaposi’s sarcoma and non-Hodgkin lymphoma while the most frequent non-AIDS malignancy was lung cancer. Malignancies are important causes of morbidity and mortality among HIV infected individuals. Due to declining mortality from AIDS-related infections, malignancies account for a growing fraction of deaths [27]. Advanced HIV infection is characterized by profound immunodeficiency which causes loss of immune control of oncogenic viruses, specifically human herpes simplex virus 8 and Epstein Barr virus [28], [29], and a large fraction of people with AIDS-associated non-Hodgkin lymphoma still die from their malignancy early in the course of the disease [30].

Our study has strengths and limitations that are worth mentioning. A strength was to the use of a standardized method of assessing the causal link between a disease or condition and death, namely the CoDe method [31]. The CoDe method was used for all deaths evaluated in the present study thus minimizing information bias that could arise from different methods and/or clinicians when assigning a cause of death. Also, all efforts were made to verify each patient’s vital status, including the verification of charts, active contact with individuals or family members and linkage with the Rio de Janeiro Mortality database using a previously validated algorithm. However, it is possible the complete ascertainment of deaths was not achieved. Another limitation refers to how representative the study population is of the HIV/AIDS patients cared for at other centers of the country. As mentioned above, IPEC provides primary, specialty, and tertiary care for HIV infected individuals and AIDS patients in Rio de Janeiro, being the largest HIV care provider in Rio de Janeiro State. The quality and level of care provided resembles that of other university hospitals and/or research-based HIV centers of the country. These centers, taken together, are responsible for specialty and tertiary cares which are services not provided at the many primary care facilities. In addition, it is located in the Southeast part of Brazil which is the region with most resources and the majority of the HIV/AIDS patients. Despite the peculiarities, HIV patients cared for at IPEC can be taken as representative of the HIV/AIDS population cared for at reference centers of Brazil.

Our results show a dramatic decreasing trend in the mortality rates from AIDS-related causes of death among HIV-infected patients from Rio de Janeiro, Brazil. It also shows that, in recent years, similar rates of death from AIDS-related and non-AIDS-related causes were observed. This finding evidences the complexity of HIV-infected patient management that is now clearly beyond the scope of care traditionally provided in most HIV specialized services and that calls for an integrated approach to treatment and prevention. Estimates of the burden of non-AIDS co-morbidities in populations affected by HIV and evidence from effective care delivery models would improve planning capabilities for better use of scarce resources. Furthermore, HIV testing targeting populations at increased risk coupled with linkage to care and early initiation of HAART are now, ever more, the cornerstone of the fight against HIV/AIDS. As for Brazil, this dramatic decrease of AIDS related causes of death happens in the midst of the country’s demographical and epidemiological transitions, where the burden of communicable and non-communicable diseases co-exist [32]. This context presents considerable challenges for the country’s public health system and should be taken into account when planning the provision of health services for the HIV-infected population.
